# Prevalence of interarm blood pressure difference is notably higher in women; the Viborg population-based screening program (VISP)

**DOI:** 10.1186/s12889-024-19388-8

**Published:** 2024-07-12

**Authors:** Marie Dahl, Jesper Winkler Andersen, Jes Lindholt, Nikolaj Thure Krarup, Britt Borregaard, Nikolai Uberg, Annette Høgh

**Affiliations:** 1 Vascular Research Unit, Department of Surgery, Viborg Regional Hospital, Toldbodgade 12, Viborg, 8800 Denmark; 2 Department of Clinical Medicine, Aarhus University, Palle Juul-Jensens Blvd. 82, Aarhus, 8200 Denmark; 3 Department of Cardiac, Thoracic and Vascular Surgery, Odense University Hospital, JB Winsløwsvej 4, Odense, 5000 Denmark; 4 Department of Clinical Research, University of Southern Denmark, Campusvej 55, Odense, 5230 Denmark; 5 Department of Cardiothoracic and Vascular Surgery, Elite Centre of Individualized Treatment of Arterial Diseases (CIMA), Clinical Institute, Odense University Hospital, University of Southern Denmark, Odense, Denmark; 6 Department of Cardiology, Viborg Regional Hospital, Heibergs Alle 5A, Viborg, 8800 Denmark; 7 Department of Cardiology, Odense University Hospital, JB Winsløwsvej 4, Odense, 5000 Denmark

**Keywords:** Systolic blood pressure difference, Interarm measurement, Cardiovascular disease, Cardiovascular risk assessment, Sex

## Abstract

**Background:**

Bilateral blood pressure (BP) measurement is important in cardiovascular prevention for identifying systolic interarm BP difference (IAD) and hypertension. We investigated sex-stratified IAD prevalence and its associations and coexistence with screen-detected peripheral atherosclerosis and hypertension. Furthermore, we determined the proportion misclassified as non-hypertensive when using the lower versus the higher reading arm.

**Methods:**

This sub-study formed part of the Viborg Screening Program (VISP), a cross-sectorial population-based cardiovascular screening programme targeting 67-year-old Danes. VISP includes screening for peripheral atherosclerosis (lower extremity arterial disease and carotid plaque), abdominal aortic aneurysm, hypertension, diabetes mellitus, and cardiac disease. Self-reported comorbidities, risk factors, and medication use were also collected. Among 4,602 attendees, 4,517 (82.1%) had eligible bilateral and repeated BP measurements. IAD was defined as a systolic BP difference ≥ 10 mmHg. IAD-associated factors (screening results and risk factors) were estimated by logistic regression; proportional coexistence was displayed by Venn diagrams (screening results).

**Results:**

We included 2,220 women (49.2%) and 2,297 men (50.8%). IAD was more predominant in women (26.8%) than men (21.0%) (*p* < 0.001). This disparity persisted after adjustment [odds ratio (OR) 1.53; 95% confidence interval (CI) 1.32–1.77]. No other association was recorded with the conditions screened for, barring potential hypertension: BP 140–159/90–99 mmHg (OR 1.68, 95% CI 1.44–1.97) and BP ≥ 160/100 mmHg (OR 1.82, 95% CI 1.49–2.23). Overall, IAD and BP ≥ 160/100 mmHg coexistence was 4% in women and 5% in men; for BP ≥ 140/90 mmHg, 13% and 14%, respectively. Among those recording a mean BP ≥ 140/90 mmHg in the higher reading arm, 14.5% of women and 15.3% of men would be misclassified as non-hypertensive compared with the lowest reading arm.

**Conclusion:**

Female sex was an independent factor of IAD prevalence but not associated with other arterial lesions. Approximately 15% needed reclassification according to BP ≥ 140/90 mmHg when the lower rather than the higher reading arm was used; verifying bilateral BP measurements improved detection of potential hypertension. In future, the predictive value of sex-stratified IAD should be assessed for cardiovascular events and death to verify its potential as a screening tool in population-based cardiovascular screening.

**Trial registration for VISP:**

NCT03395509:10/12/2018.

**Supplementary Information:**

The online version contains supplementary material available at 10.1186/s12889-024-19388-8.

## Background

In cardiovascular prevention, identifying at-risk individuals is crucial to address modifiable cardiovascular risk factors. Prophylactic treatment and lifestyle modifications reduce the risk of fatal and non-fatal major adverse cardiovascular events (MACE) associated with high blood pressure (BP) [1]. Individual-level data from 1.5 mill. persons in 112 cohorts showed that the ten-year incidence of cardiovascular disease (CVD) was higher among individuals with raised systolic BP than among individuals with other modifiable risk factors (e.g., smoking and diabetes), particularly in women (29.3%), slightly less in men (21.6%) [2].

International guidelines stress the importance of measuring BP in both arms to diagnose and manage hypertension accurately. Monitoring is recommended on the higher reading arm when a difference is detected [3–5]. Bilateral BP measurements are also important in detecting systolic interarm blood pressure difference (IAD). In a recent meta-analysis with 53,827 individuals, Clark et al. reported that IAD ≥ 10 mmHg was associated with increased MACE and all-cause mortality [6]. Moreover, in a second meta-analysis by Clark et al., BP measured in the higher versus the lower reading arm was superior in predicting MACE and avoiding misclassification of BP [7]. Thus, bilateral BP readings are of clinical relevance and substantial interest for preventive purposes. Despite this, bilateral BP measurements are infrequently obtained [8].

In the Danish “Viborg Screening Program” (VISP) [9], a population-based screening programme monitoring multiple cardiovascular conditions in 67-year-olds, bilateral BP readings are the standard procedure for identifying the arm with the higher reading for subsequent BP readings. Those with a verified BP ≥ 160/100 mmHg initiate antihypertensive medication treatment according to the global International Society of Hypertension Global Hypertension Practice Guidelines [3]. In VISP, BP ≥ 160/100 mmHg was observed in 13.8% of women and 17.1% of men [10]. However, the sex-stratified IAD prevalence, its associations and coexistence with other conditions and the relevance of using the higher versus the lower reading arm remain unclarified in population-based screening. Thus, we aimed to describe (1) the sex-stratified prevalence of systolic IAD ≥ 10 mmHg in the VISP cohort and (2) determine its association and coexistence with associated screen-detected conditions while (3) reporting the proportion misclassified as non-hypertensive when using the lower versus the higher reading arm.

## Methods

### Study population and setting

This Danish study was a non-predefined sub-study adopting a cross-sectional design using data from VISP, previously reported in detail [9]. Briefly, screening participants were invited on their 67th birthday without exclusions, and the programme included examinations for peripheral atherosclerosis (lower extremity arterial disease (LEAD) and carotid plaque (CP)), abdominal aortic aneurysm (AAA), hypertension, cardiac arrhythmia or ischaemia, and diabetes mellitus. Diagnostic criteria and follow-up in VISP are presented in Table 1. In addition to the clinical examinations, participants self-reported risk factors, morbidity and prescribed medicine were collected. The Viborg Healthcare Centre hosts VISP within a collaborative setting, including primary and secondary healthcare. The first participants were enrolled in August 2014, and enrolment is still ongoing. During the initial five years, 5,505 participants were invited, 4,602 of whom participated and subsequently constituted the study population. For further details, please refer to the study protocol [9].

**Table 1:** Diagnostic criteria and follow-up in the Viborg Screening Program (VISP)

Diagnostic criteria		Follow-Up, in case of positive screening result
Carotid plaque	Focal structure protruding into the lumen ≥ 0.5 mm or ≥ 50% of the vessel diameter.	Recommendation of lipid-lowering and antiplatelet therapy along with lifestyle modification counselling.
Lower extremity arterial disease	Ankle-brachial index < 0.9 or ≥ 1.4.	Recommendation of lipid-lowering and antiplatelet therapy along with lifestyle modification counselling.
Aortic ectasia or aneurysm	Aortic diameter of ≥ 25 mm and ≥ 30 mm as measured by B-mode ultrasonography.	Participants with ectasia were offered a re-screening after 5 years. Participants with aneurysm were recommended lipid-lowering and antiplatelet therapy, lifestyle modification counselling and follow-up imaging according to size: 30–49 mm: annual ultrasound scan. ≥ 50 mm: CT scan and vascular surgical consultation.
Hypertension	BP ≥ 160/100 mmHg in the higher reading arm.	Recommendation of three-day home BP measurement for verification of hypertension including follow-up in VISP or by their general practitioner.
Arrhythmia and ischaemia	Significant changes in a single 12-lead electrocardiogram, assessed by an expert cardiologist.	Referred for follow-up by an expert cardiologist.
Diabetes	HbA1c ≥ 48 mmol/mol.	Participants without previously known diabetes and HbA1c ≥ 48 mmol/mol were referred for follow-up by their general practitioner.

Abbreviations: BP, blood pressure; CT, computer tomography; HbA1c, glycated haemoglobin

### Data sources

#### Self-reported data

In this study, selected information from the questionnaire attached to the VISP invitation letter was used to obtain data on lifestyle factors (smoking, alcohol consumption and physical activity), weight and height used to calculate Body Mass Index (BMI, kg/m^2^), and medication use. Smoking habits were collected in three categories: never, former or current smokers. Alcohol consumption was categorised according to the recommendation of the Danish Health Authority at the time into low risk (women < 7 units/week and men < 14 units/week) and high risk (women > 14 units/week and men > 21 units/week). Level of physical activity was grouped as low (sedentary recreational activities like reading and watching television), moderate (low-intensity physical activity, at least 4 h a week), high (high-intensity activity, at least 4 h a week) and very high (competitive sports regularly) during the past year, in accordance with the Danish Regional Health Survey [11]. BMI was subdivided into three groups: BMI < 25, 25–29 and ≥ 30. Use of medicine was divided into four groups: lipid-lowering agents, antiplatelet (aspirin, clopidogrel), antihypertensive (angiotensin-converting enzyme (ACE) inhibitors, angiotensin II receptor blockers, calcium channel blockers, thiazides) and antidiabetic agents (insulin and oral antidiabetic agents). The questionnaire developed for VISP is available in Supplementary Material 1; which also includes the EuroQoL five-dimension instrument and the Walking Impairment Questionnaire.

#### Information on sex

Information about sex was generated from the participants’ unique 10-digit civil registration number (CPR) provided by the Central Office of Civil Registration System [12].

#### Blood pressure measurement

BP was measured three times in total per screening participant, all in supine position, using the oscillometric method with appropriate cuff size as recommended for use in clinical practice [5, 13]. The Microlife BP A6 PC devices were used with a grade A/A accuracy upon validation in accordance with the protocol of the British and Irish Hypertension Society. First, BP was measured simultaneously and bilaterally immediately after cuff placement, a durable approach in accordance with the Best Rest Trial [14], with identical monitors on the left and right arm. The second and third measurements were made in connection with an ankle-brachial index (ABI) assessment with the participant in a supine position; these two measurements were obtained unilaterally on the arm recording the highest systolic BP at the first measurement.

IAD was calculated from the first BP measurement by subtracting the lowest from the highest systolic BP. IAD was defined as an interarm BP difference ≥ 10 mmHg and sub-grouped into IAD ≥ 10 to 19 mmHg, ≥ 20 to 29 mmHg and ≥ 30 mmHg.

BP misclassification was based on the lower versus the higher reading arm upon the first BP measurement using the thresholds recommended in the International Society of Hypertension guideline; 140–159/90–99 mmHg and ≥ 160/100 mmHg [3]. In Venn diagrams, BP was categorised by means as BP ≥ 140/90 mmHg and BP ≥ 160/100 mmHg. Mean BP was calculated as the mean of the second and third BP measurements; the highest systolic or diastolic value of the mean measurement determined the BP categorisation.

Bilateral brachial BP was used to calculate the ABI; by dividing the mean of the systolic BPs in the tibialis posterior and dorsalis pedis arteries by the higher of the two systolic brachial BPs.

### Statistical analysis

Baseline characteristics, medication and screening results were stratified by IAD status (< 10 mmHg or ≥ 10 mmHg) and presented as categorical variables with absolute numbers, proportion and related 95% confidence intervals (CI). Furthermore, sex-stratified results were reported for those with IAD > 10 mmHg. In sub-analysis, IAD was expressed as mean ± standard deviation (SD). Categorical and continuous variables were analysed by Pearson’s chi-squared test and Student’s *t*-test, respectively.

Unadjusted and adjusted logistic regression analyses were used to investigate factors associated with IAD ≥ 10 mmHg, reported as odds ratio (OR) with 95% CI. Analyses included observations with complete data. In the adjusted analyses, we entered variables with an unadjusted *p*-value of < 0.1 and smoking as smoking may constitute a clinically relevant risk factor for IAD [15, 16]. To finally indicate statistical significance, a *p* < 0.05 was used.

Venn diagrams were applied to display the proportional coexistence of IAD with other screening conditions, but only for screening results positively associated with IAD in adjusted logistic regression analyses.

In accordance with the General Data Protection Regulation, the web-based Research Electronic Data Capture (REDCap) system hosted at Open Patient Data Explorative Network, Odense University Hospital, Denmark, was used for storage and processing of all data. STATA version 18.0 (StataCorp, College Station, TX, USA) was used to perform the statistical analyses.

## Results

**Fig. 1:**
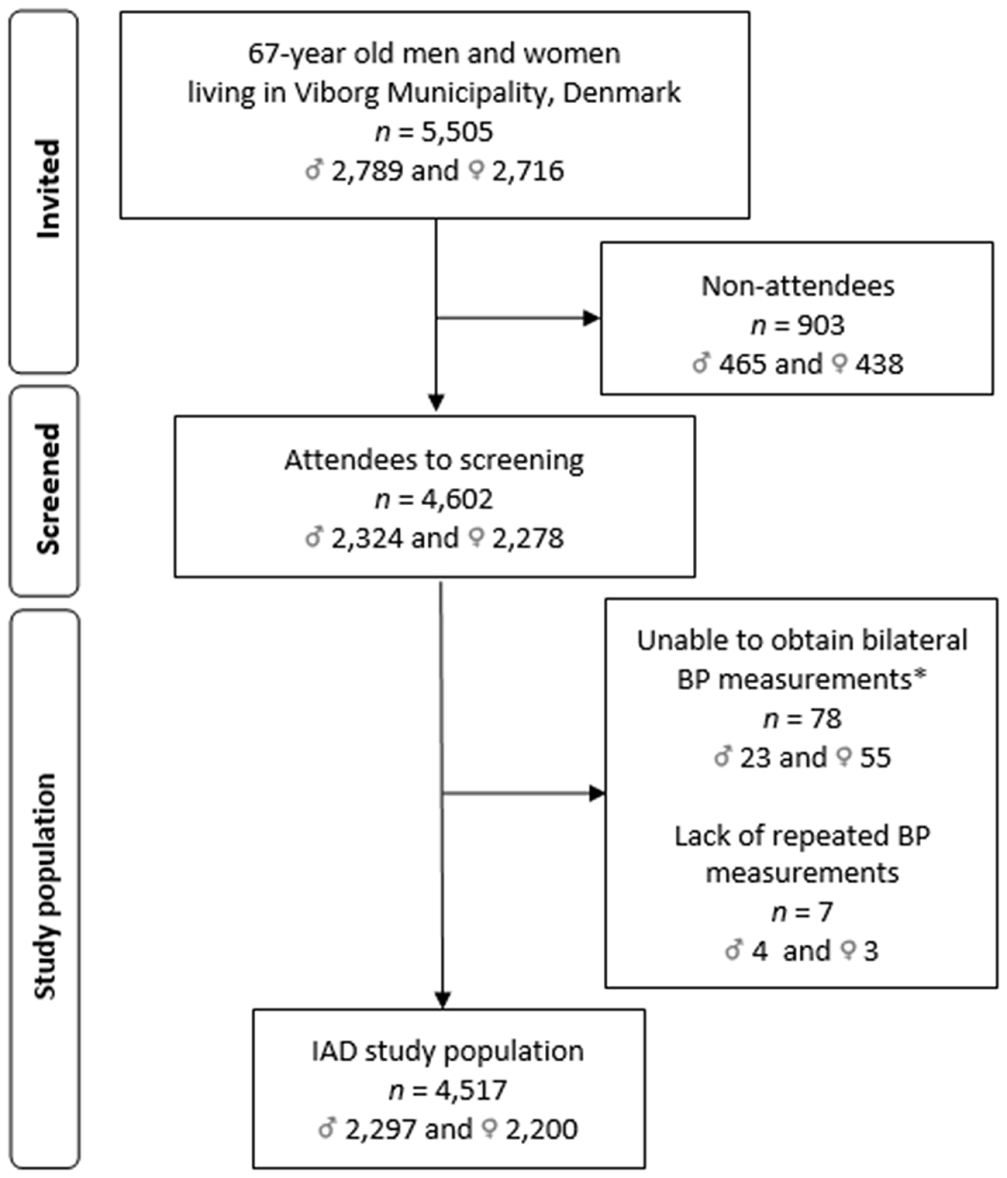
Flow diagram of the study population. Abbreviation: IAD, interarm blood pressure difference. *Causes of lacking bilateral BP measurement: discomfort or abstained from bilateral BP measurement due to previous breast cancer and surgical mastectomy leaving the affected side inappropriate for BP readings

During the first five years of enrolment, 5,505 men and women were invited to participate in VISP. In total, 83.6% (*n* = 4,602) accepted the invitation with no sex difference in participation rates. A total of 1.8% of the participants were excluded due to a lack of bilateral (*n* = 78) and repeated BP measurements (*n* = 7), leaving 82.1% (*n* = 4,517) of the participants eligible for the study (Fig. 1).

Lifestyle habits like smoking and alcohol consumption were similar for participants with and without IAD ≥ 10 mmHg, whereas the level of physical exercise tended to be different (*p* = 0.058). Among participants with an IAD ≥ 10 mmHg, a significantly higher proportion of participants were obese; 29.6% versus 19.7% among those with an IAD < 10 mmHg (*p* < 0.001) (Table 2).

**Table 2:** Characteristics of participants by IAD status (*n* = 4,517)

IAD, mmHg	Missing, *n*	Total, *n*	IAD < 10 mmHg		IAD ≥ 10 mmHg	
			*n* = 3,440	95% CI	*n* = 1,077	95% CI
**Sex**	0					
Women		2,220 (49.2)	1,625 (47.2)	45.57–48.91	595 (55.3)	52.26–58.19
Men		2,297 (50.8)	1,815 (52.8)	51.09–54.43	482 (44.8)	41.81–47.74
**Lifestyle habits**						
**Smoking**	26					
Never		1,855 (41.3)	1,419 (41.5)	39.85–43.15	436 (40.7)	37.80-43.15
Current		737 (16.4)	560 (16.4)	15.17–17.65	177 (16.5)	14.42–18.87
Former		1,899 (42.3)	1,441 (42.1)	40.49–43.80	458 (42.8)	39.83–45.75
**Alcohol**, units/week	227					
♀: <7, ♂: <14		3,422 (79.8)	2,585 (79.4)	77.97-80-75	837 (81.0)	78.44–83.23
♀: 7–14, ♂: 14–21		595 (13.9)	454 (13.9)	12.80-15.18	141 (13.6)	11.68–15.87
♀: ≥14, ♂: ≥21		273 (6.3)	217 (6.7)	5.86–7.57	56 (5.4)	4.19–6.97
Level of exercise*	45					
Low		418 (9.4)	301 (8.8)	7.93–9.84	117 (11.0)	9.23-13.00
Moderate		2,832 (63.3)	2,142 (62.9)	61.25–64.50	690 (64.7)	61.81–67.54
High		1,153 (25.8)	909 (26.7)	25.23–28.20	244 (22.9)	20.46–25.51
Very high		69 (1.5)	54 (1.6)	1.22–2.06	15 (1.4)	0.85–2.32
**BMI**, kg/ m^2^	57					
< 25		1,698 (38.1)	1,370 (40.3)	38.67–41.97	328 (30.9)	28.20-33.76
25–30		1,780 (39.9)	1,361 (40.0)	38.41–41.70	419 (39.5)	36.59–42.47
≥ 30		982 (22.0)	668 (19.7)	18.35–21.02	314 (29.6)	26.92–32.41
**Medications**						
Lipid-lowering	25	1,380 (30.7)	1,050 (30.7)	29.16–32.25	330 (30.8)	28.14–33.68
Antiplatelet	25	834 (18.6)	644 (18.8)	17.54–20.16	190 (17.8)	15.58–20.16
Antihypertensive	25	1,855 (41.3)	1,410 (41.2)	39.56–42.86	445 (41.6)	38.67–44.57
Antidiabetic	25	345 (7.7)	257 (7.5)	6.67–8.44	88 (8.2)	6.72–10.03
**Screening results**						
Peripheral atherosclerosis Carotid plaque	8 n/a**	1,802 (40.0) 1,735 (38.4)	1,346 (39.2) 1,296 (37.7)	37.55–40.82 36.07–39.31	456 (42.5) 439 (40.8)	39.57–45.48 37.90-43.77
LEAD	7	249 (5.5)	176 (5.1)	4.43–5.91	73 (6.8)	5.44–8.47
BP 140–159/90–99 mmHg	0	1,463 (32.4)	1,047 (30.4)	28.92-32.00	416 (38.6)	35.76–41.57
BP ≥ 160/100, mmHg	0	660 (14.6)	458 (13.3)	12.22–14.49	202 (18.8)	16.53–21.20
HbA1c ≥ 48, mmol/mol	n/a**	404 (8.9)	296 (8.6)	7.72–9.60	108 (10.0)	8.37–11.97

Abbreviations: BMI, Body Mass Index; BP, blood pressure; CI, confidence interval; HbA1c, glycated haemoglobin; IAD, interarm blood pressure difference; LEAD, lower extremity arterial disease

* Level of physical activity was grouped into: low (sedentary recreational activities like reading and watching television), moderate (low-intensity physical activity, at least 4 h a week), high (activity of high intensity, at least 4 h a week) and very high (competitive sports on a regular basis) during the past year

** To protect anonymity, we are not allowed to report numbers ≤ 3

### Systolic interarm blood pressure difference

In total, 1,077 (23.8%) of the 4,517 participants had an IAD ≥ 10 mmHg (95% CI 22.61–25.11), with a higher frequency being recorded in women (26.8%) than men (21.0%) (*p* < 0.001). This female preponderance was observed in all IAD subgroups (Figure S1, Supplementary Material 2). In participants with IAD < 10 mmHg, the mean IAD was 3.98 (2.63) compared with 15.22 (5.86) in those with IAD ≥ 10 mmHg. No BP difference was observed in 5.4%. Among those with an IAD ≥ 10 mmHg, BP was most frequently lowest in the left arm (59.0% versus 41.0%) (*p* < 0.001).

Among the positive screening results, hypertension was significantly more frequent in those with IAD ≥ 10 mmHg (BP 140–159/90–99, 38.6%; BP ≥ 160/100, 18.8%) than in those with IAD < 10mmHg (BP 140–159/90–99, 30.4%; BP ≥ 160/100, 13.3%) (*p* < 0.001) (Table 2).

Table 3 presents the characteristics of participants with IAD ≥ 10 mmHg stratified by sex. The table shows a significantly higher rate of peripheral atherosclerosis (LEAD and CP) in men (49.7%) than in women (36.7) (*p* < 0.001). Table 3 also shows that approximately 30% were taking lipid-lowering medication before participating in VISP (no gender-specific difference), whereas only 16.6% of women and 19.2% of men were in antiplatelet therapy. Use of antihypertensive medication was frequent in both men and women, with IAD ≥ 10, 40.6% and 42.8%, respectively.

**Table 3:** Characteristics of participants with an IAD ≥ 10 mmHg, by sex (*n* = 1,077)

Sex	Missing		Total, *n*	Women		Men	
	0			*n* = 595	95% CI	*n* = 482	95% CI
**Lifestyle habits**							
**Smoking**	6						
Never			436 (40.7)	264 (44.7)	40.70-48.71	172 (35.8)	31.66–40.23
Current			177 (16.5)	91 (15.4)	12.70-18.54	86 (17.9)	14.73–21.61
Former			458 (42.8)	236 (39.9)	36.05–43.94	222 (46.3)	41.83–50.73
**Alcohol**, units/week	43						
♀: <7, ♂: <14			837 (81.0)	470 (82.5)	79.11–85.37	367 (79.1)	75.15–82.56
♀: 7–14, ♂: 14–21			141 (13.6)	81 (14.2)	11.57–17.33	60 (12.9)	10.17–16.31
♀: ≥14, ♂: U ≥ 21			56 (5.4)	19 (3.3)	2.13–5.17	37 (8.0)	5.83–10.82
Level of exercise*	11						
Low			117 (11.0)	60 (10.2)	8.00-12.93	57 (11.9)	9.31–15.15
Moderate			690 (64.7)	405 (68.9)	65.01–72.50	285 (59.6)	55.15–63.94
High			244 (22.9)	119 (20.2)	17.18–23.68	125 (26.2)	22.40-30.28
Very high			15 (1.4)	4 (0.7)	0.26–1.80	11 (2.3)	1.28–4.11
**BMI**,** kg/m**^**2**^	16						
< 25			328 (30.9)	204 (35.1)	31.28–39.02	124 (25.9)	22.16-30.00
25–30			419 (39.5)	206 (35.4)	31.61–39.37	213 (44.5)	40.07–48.96
≥ 30			314 (29.6)	172 (29.5)	25.98–33.39	142 (29.6)	25.72–33.90
**Medications**							
Lipid-lowering	7		330 (30.8)	185 (31.3)	27.69–35.16	145 (30.3)	26.32–34.54
Antiplatelet	7		190 (17.8)	98 (16.6)	13.79–19.81	92 (19.2)	15.92–22.99
Antihypertensive	7		445 (41.6)	240 (40.6)	36.71–44.63	205 (42.8)	38.43–47.28
Antidiabetic	7		88 (8.2)	50 (8.5)	6.47–10.99	38 (7.9)	5.82–10.72
**Screening results**							
Peripheral atherosclerosis**	4		456 (42.5)	217 (36.7)	32.86–40.62	239 (49.7)	45.23–54.15
BP 140–159/90–99 mmHg	0		416 (38.6)	196 (32.9)	29.28–36.83	220 (45.6)	41.24–50.12
BP ≥ 160/100, mmHg	0		202 (18.8)	95 (16.0)	13.23–19.14	107 (22.2)	18.71–26.13
HbA1c ≥ 48, mmol/mol	0		108 (10.0)	61 (10.3)	8.06–12.96	47 (9.8)	7.40-12.74

Abbreviations: BMI, Body Mass Index; BP, blood pressure; CI, confidence interval; HbA1c, glycated haemoglobin; IAD, interarm blood pressure difference

* Level of physical activity was grouped into: low (sedentary recreational activities like reading and watching television), moderate (low-intensity physical activity, at least 4 h a week), high (activity of high intensity, at least 4 h a week) and very high (competitive sports on a regular basis) during the past year

** Peripheral atherosclerosis constitutes lower extremity arterial disease and carotid plaque

Table 4 displays factors associated with an IAD ≥ 10 mmHg. After adjusting for smoking habits, level of exercise, BMI and screening results, the predominant female findings of IAD ≥ 10 mmHg persisted (OR 1.53, 95% CI 1.32–1.77, *p* < 0.001). Furthermore, BMI was associated with IAD ≥ 10 mmHg; BMI ≥ 25–29 (OR 1.32, 95% CI 1.11–1.56, *p =* 0.001) and BMI ≥ 30 (OR 1.88, 95% CI 1.55–2.27, *p* < 0.001) when using BMI < 25 as a reference. Among positive screening results, only raised BP was associated with IAD ≥ 10 mmHg; BP 140–159/90–99 (OR 1.68, 95% CI 1.43–1.97, *p* < 0.001) and BP ≥ 160/100 (OR 1.82, 95% CI 1.49–2.23, *p* < 0.001) (Table 4), leaving no associations with diabetes or peripheral atherosclerosis such as CP and LEAD.

**Table 4:** Factors associated with an IAD ≥ 10 mmHg

	Unadjusted			Adjusted*		
	OR	95% CI	*P*-value	OR	95% CI	*P*-value
**Sex**						
Men	*ref*			*ref*		
Women	1.38	1.20–1.58	< 0.001	1.53	1.32–1.77	< 0.001
**Lifestyle habits**						
**Smoking**						
Never	*ref*			*ref*		
Current	1.03	0.84–1.26	0.782	1.10	0.89–1.36	0.388
Former	1.03	0.89–1.20	0.695	1.05	0.90–1.23	0.31
**Alcohol**, units/week						
♀: <7, ♂: <14	*ref*					
♀: 7–14, ♂: 14–21	0.96	0.78–1.18	0.686	-		
♀: ≥14, ♂: ≥21	0.80	0.59–1.08	0.143	-		
**Level of exercise**						
Low	*ref*			*ref*		
Moderate	0.83	0.66–1.04	0.110	0.90	0.71–1.14	0.386
High	0.69	0.53–0.89	0.005	0.84	0.64–1.11	0.221
Very high	0.71	0.39–1.32	0.281	0.97	0.52–1.82	0.934
**BMI**,** kg/m**^**2**^						
< 25	*ref*			*ref*		
25–30	1.29	1.09–1.51	0.002	1.32	1.11–1.56	0.001
≥ 30	1.96	1.64–2.35	< 0.001	1.88	1.55–2.27	< 0.001
**Lipid-lowering**						
Not using	*ref*					
Using	1.00	0.87–1.17	0.922	-		
**Antiplatelet**						
Not using	*ref*					
Using	0.93	0.78–1.11	0.435	-		
**Antihypertensive**						
Not using	*ref*					
Using	1.02	0.88–1.17	0.823	-		
**Antidiabetic**						
Not using	*ref*					
Using	1.10	0.86–1.42	0.444	-		
Peripheral atherosclerosis **						
Not found	*ref*			*ref*		
Found	1.15	1.00-1.32	0.052	1.11	0.95–1.28	0.191
**BP ≥ 160/100**,** mmHg** ***						
Not found	*ref*			*ref*		
140–159/90–99	1.68	1.44–1.95	< 0.001	1.68	1.44–1.97	< 0.001
≥ 160/100 mmHg	1.86	1.53–2.26	< 0.001	1.82	1.49–2.23	< 0.001
**HbA1c ≥ 48**,** mmol/mol**						
Not found	*ref*					
Found	1.18	0.94–1.49	0.156	-		

Abbreviations: BMI, Body Mass Index; BP, blood pressure; CI, confidence interval; HbA1c, glycated haemoglobin; IAD, interarm blood pressure difference. OR, odds ratio

* In the adjusted analyses, we entered variables with an unadjusted p value of < 0.1 and smoking; as smoking may constitute a clinically relevant risk factor for interarm difference

** Peripheral atherosclerosis constitutes lower extremity arterial disease and carotid plaque

*** Blood pressure is the mean of the second and third blood pressure measurements in accordance with guidelines

### Coexistence of IAD and screen-detected hypertension

**Fig. 2:**
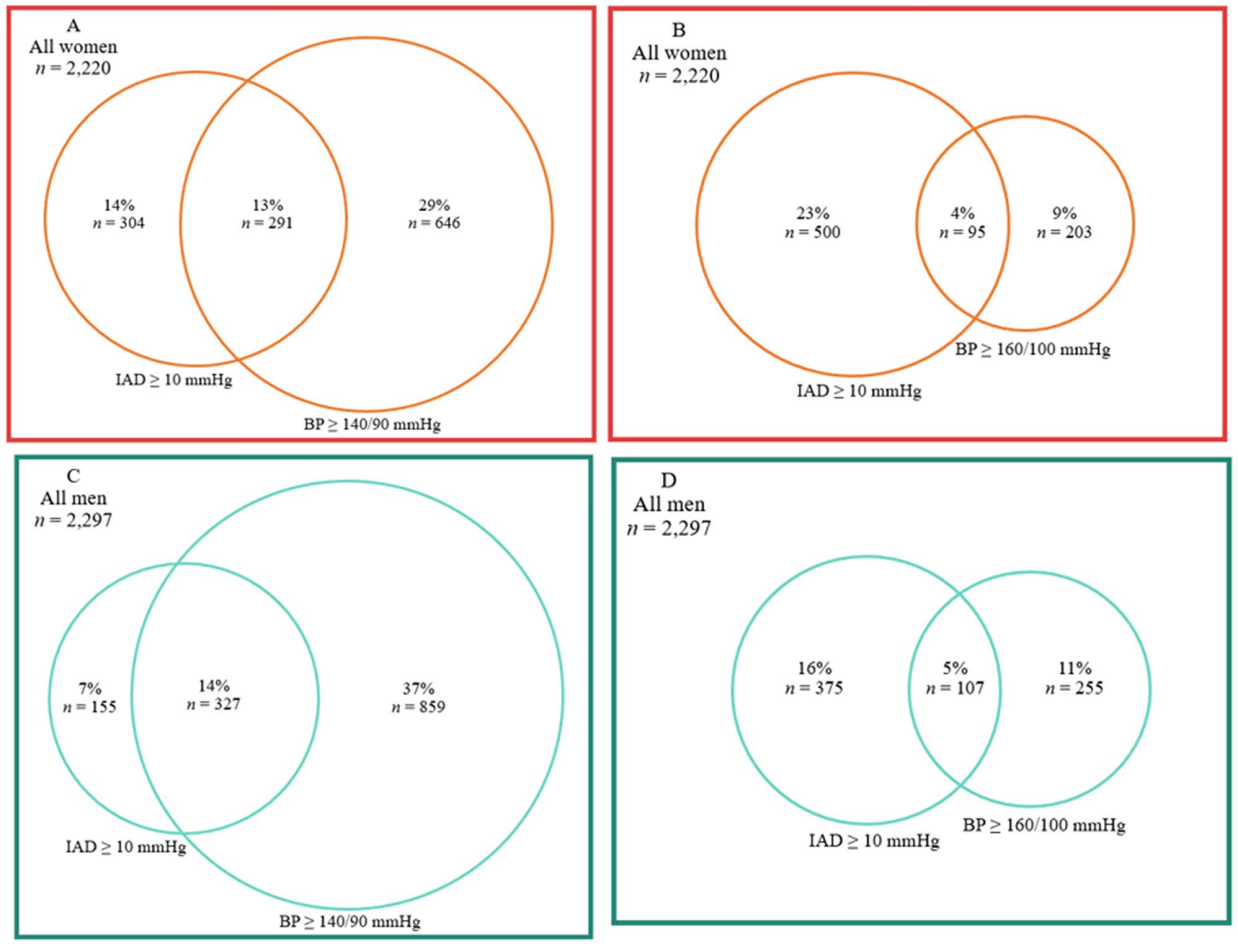
Venn diagram displaying coexistence of IAD ≥ 10 mmHg and BP at 140/90 mmHg or 160/100 mmHg thresholds, stratified by sex. Abbreviations: BP, blood pressure; IAD, interarm blood pressure difference. Percentages are for all women (**A** and **B**) or men (**C** and **D**), respectively

In the entire screening cohort, the coexistence of IAD ≥ 10 mmHg and BP ≥ 140/90 mmHg was observed in 13% of all women. A similar tendency was seen in all men (14%). Coexistence of IAD and BP ≥ 160/100 mmHg was 4% in women and 5% in men (Fig. 2).

In the participants with IAD ≥ 10 mmHg or/and BP ≥ 140/90 mmHg, coexistence of IAD increased to 23% in women and 24% in men. At a BP threshold of at least 160/100 mmHg, coexistence of IAD decreased to 12% in women and 15% in men (Figure S2, Supplementary Material 2).

### Higher versus lower reading arm for classification of hypertension

For both sexes, differences in the proportions of high BP were found when comparing the higher and lower reading arm (Table 5). At the 140–159/90–99 mmHg BP threshold, 6.2% of women and 5.3% of men would have been misclassified when using the lower rather than the higher readings (women: 32.4% minus 26.2% and men: 38.4% minus 33.1%). For BP ≥ 160/100 mmHg, misclassification rates were 8.3% in women (22.6 minus 14.3) and 10.0% in men (27.0 minus 17.0%), resulting in a total misclassification of 14.5% in women and 15.3% in men.

**Table 5:** Prevalence of screen-detected hypertension, stratified by sex

BP, mmHg	Total, *n*	Women *n* = 2,220		Men *n* = 2,297	
		*n* (%)	95% CI	*n* (%)	95% CI
First systolic BP reading*					
**Higher reading arm**					
< 140	1,896 (42.0)	1,036 (46.7)	44.60-48.75	860 (37.4)	35.48–39.44
140–159	1,548 (34.3)	692 (31.2)	29.28–33.13	856 (37.3)	35.31–39.26
≥ 160	1,073 (23.7)	492 (22.1)	20.48–23.94	581 (25.3)	23.56–27.11
**Lower reading arm**					
< 140	2,550 (56.5)	1,351 (60.9)	58.81–62.87	1,199 (52.2)	50.15–54.24
140–159	1,280 (28.3)	556 (25.0)	23.29–26.89	724 (31.5)	29.65–33.45
≥ 160	687 (15.2)	313 (14.1)	12.71–15.61	374 (16.3)	14.83–17.85
First systolic and/or diastolic BP reading*					
**Higher reading arm**					
< 140 / 90	1,793 (39.7)	998 (45.0)	42.90-47.03	795 (34.6)	32.69–36.58
140–159 /90–99	1,600 (35.4)	719 (32.4)	30.47–34.36	881 (38.4)	36.39–40.36
≥ 160 and/or ≥ 100	1,124 (24.9)	503 (22.6)	20.96–24.45	621 (27.0)	25.26–28.89
**Lower reading arm**					
< 140 / 90	2,466 (54.6)	1,320 (59.5)	57.40-61.48	1,146 (49.9)	47.85–51.94
140–159 / 90–99	1,343 (29.7)	582 (26.2)	24.43–28.09	761 (33.1)	31.23–35.08
≥ 160 / 100	708 (15.7)	318 (14.3)	12.93–15.84	39 (17.0)	15.50-18.57
Second and third systolic and/or diastolic BPs in higher reading arm*					
**Mean**					
< 140 / 90	2,394 (53.0)	1,283 (57.8)	55.73–59.83	1,111 (48.4)	46.33–50.41
140–159 / 90–99	1,463 (32.4)	639 (28.8)	26.94–30.70	824 (35.9)	33.94–37.86
≥ 160 / 100	660 (14.6)	298 (13.4)	12.07–14.91	362 (15.7)	14.33–17.31

Abbreviations: BP, blood pressure; CI, confidence interval

* Missing, *n* = 0

A comparison of the first BP measurements showed similar proportions classified as hypertensive, regardless of whether their systolic reading alone or both their systolic and diastolic readings were considered (Table 5).

## Discussion

In this population-based cross-sectional study of 4,517 Danes aged 67 years, we investigated sex differences in IAD prevalence and its associations and coexistence with screen-detected cardiovascular conditions. Finally, we studied the magnitude of hypertension misclassification. Overall, we found a 23.8% prevalence of IAD ≥ 10 mmHg. The prevalence of systolic IAD is reported to fluctuate with age, ethnicity and cohort comorbidity. A meta-analysis by Clark et al. found a pooled 11.2% prevalence of IAD ≥ 10 mmHg in patients from primary care populations [17]. However, in cross-sectional studies, IAD prevalences reached up to 19% among primary care patients with hypertension (mean age 69.6 years) [18] and up to 27% among 18-60-year-old healthy male Indians [19]. Among 3,350 asymptomatic men and women with elevated CVD risk (mean age 61.9 years) enrolled in a randomised trial within primary care, IAD was observed in 38% [20]. In secondary care, IAD prevalence was observed in up to 48% of patients with coronary artery disease [21]. Among Danes referred to a vascular laboratory for potential LEAD, of whom 46.8% were diagnosed with LEAD, 27% had IAD ≥ 10 mmHg at three repeated simultaneous BP measurements [22].

Previous studies have shown that conducting multiple measurements generates fewer individuals with an IAD [23, 24]. Our observation of IAD prevalence in a general population may be attributed to initially using only a single simultaneous bilateral BP reading to calculate IAD. In contrast, our approach involved simultaneously measured BP, which is a favourable approach for identifying IAD compared with sequential bilateral BP [17].

We found that IAD reflected a significant female preponderance, a trend reflected in all IAD subgroups. This sex-related difference persisted after adjustment for relevant available cardiovascular risk factors and screening results. In contrast, no sex differences in IAD prevalence were reported in an Indian population (men 42.9%; women 44.2%) [25] or an African population (men 19%; women 20%) [26]. Moreover, in a study of 484 Finnish participants (mean age 49.7 years), a similar IAD prevalence was observed in men (12.1%) and women (8.3%) (*p =* 0.16) [27]. Thus, further investigation into sex-stratified prevalence and potential causality is warranted in a large-scale, elderly Caucasian population.

In the present study, screen-detected raised BP was the only medical condition positively associated with IAD. In contrast, other studies have reported IAD to be related to the presence of co-occurring cardiovascular conditions, e.g., LEAD, hypertension, diabetes mellitus, carotid stenosis and abdominal aortic dissection [28–30]. We chose not to report OR for LEAD separately as the sensitivity of IAD in identifying LEAD is low (15%), albeit its specificity is high (96%) [30]. These diverse findings may partly be explained by the composition of the VISP cohort, which comprises a general population of all 67-year-old persons in a restricted geographical area with a low prevalence of, e.g., LEAD (5.5%), and as we report carotid plaque and not carotid stenosis. Similarly, no association between IAD and the presence of carotid plaque was observed in a cross-sectoral study among 1,426 individuals in primary care [31]. When assessing IAD as a screening tool, we believe that data from populations with a low-to-moderate CVD risk are important. Such populations will mirror those seen in general practice.

Coexistence of IAD and BP at a threshold of ≥ 140/90 mmHg was seen in 13% of women and 14% of men. This coexistence was lower at a BP threshold of at least 160/100 mmHg, indicating that IAD was an isolated screening result. This result is novel and unexplored in the literature.

Systolic IAD has been associated with an increased risk of MACE and all-cause mortality [6]. In a sub-analysis of the Cardiovascular Outcomes for People Using Anticoagulation Strategies (COMPASS) trial, consisting of patients with chronic LEAD or coronary artery disease, a similar risk was observed when comparing those with and without IAD in terms of the composite endpoint of MACE, except for stroke [32]. Thus, IAD may constitute a risk marker in advanced risk assessment, mainly in primary prevention. A systematic Cochrane review found that frequently used cardiovascular risk scores for primary cardiovascular prevention had an uncertain effect [33]. Emerging evidence suggests that IAD may supplement existing risk prediction scores, such as Framingham and QRISK2 [6], substantiating the need for further evaluation of new CVD assessment methods. Nevertheless, in cardiovascular screening initiatives like VISP, it is important to research and investigate the additional value of including IAD as a risk marker, especially considering a gender-specific perspective. International hypertension management guidelines recognise IAD as a marker in atherosclerotic disease associated with increased CVD risk at a threshold of IAD ≥ 15 mmHg (class I, level A [5] and class I, level C [4]). However, these guidelines have not taken into account the meta-analysis by INTERPRESS-IPD (the Interarm Blood Pressure Difference Individual Patient Data Collaboration) headed by Clark, in which a threshold of IAD ≥ 10 mmHg is recommended [6]. This new evidence served as the rationale for our decision on the IAD threshold. As of now, cardiovascular prophylaxis with antiplatelet and lipid-lowering agents is not recommended for IAD. Noteworthy, identification of IAD may hold prophylactic potential in the general population. We found that only a third of the VISP participants with IAD ≥ 10 mmHg received lipid-lowering therapy before screening, and less than 20% received antiplatelet therapy. Further research is warranted to clarify the rationale for initiating such prophylactic medication upon IAD.

When adopting a mean BP threshold of ≥ 140/90 mmHg in the higher versus the lower reading arm, we found that 14.5% of women and 15.3% of men needed reclassification. Our results aligned with the results from a secondary meta-analysis by Clark et al. with multinational participation (*n* = 53,173); overall, 12% of men and women required reclassification from non-hypertensive to hypertensive when using the higher rather than the lower reading arm to diagnose hypertension at a systolic BP of 130 and 140 mmHg [7]. Moreover, Clark et al. found that utilising a higher rather than a lower reading arm is superior in predicting MACE and all-cause mortality [7]. These findings emphasise the significance of considering BP readings from both arms and favouring the measurement from the higher arm in clinical practice.

### Strengths and limitations

The main strengths of our study are its population-based design and high participation rate with a complete dataset (82.1%). Moreover, including both sexes is a strength as women continue to be underrepresented in cardiovascular research [34]. Another strength is that we used simultaneous BP measurements as sequential have been reported to overestimate IAD threefold compared with simultaneous BP measurements [17].

The limitations of our study are related to the fact that we only obtained one simultaneous BP measurement. Thus, our study cannot contribute to clarifying the reproducibility of IAD or its coexistence with CVD conditions. The Multi-Ethnic Study of Atherosclerosis (MESA) found that high systolic IAD was not persistent across three exams conducted throughout a 9.5-year follow-up period. Nevertheless, Duprez et al. found that at least one maximum absolute IAD ≥ 15 mmHg had a graded association with incident stroke and LEAD [35]. Furthermore, the review by Clark et al. suggested that a threshold for systolic IAD ≥ 10 mmHg was sufficient based on a single pair of sequential BP measurements [6]. In the VISP set-up, BP measurements were obtained as part of non-automatic ABI measurements and based on three consecutive measurements conducted during the same visit. Ideally, the reproducibility of misclassification should be assessed at separate visits. However, performing separate BP measurements jeopardises the feasibility of VISP. Additionally, it would have been preferable to perform BP measurements without the screening nurses being present to avoid any white-coat effect. On the other hand, in a review, Clark et al. concluded that white-coat hypertension is less frequently diagnosed when BP measurements are made by nurses than by doctors [36]. The white-coat effect occurs at all grades of hypertension but is greatest for grade 1 hypertension. This supports our threshold for action of BP ≥ 160/100 mmHg [4] to limit the risk of false positives. Finally, using self-reported information carries a risk of social desirability bias.

## Conclusion

This study highlighted the importance of bilateral BP measurements in identifying IAD and enhancing the diagnostic accuracy of hypertension. Female sex and raised BP emerged as an independent factor for IAD prevalence, whereas IAD showed no association with diabetes or other arterial lesions such as LEAD or CP. The coexistence of IAD and raised BP was observed in up to 14% of participants, with a decreasing tendency at a higher BP threshold, indicating that IAD may be an isolated factor in the general population. Using the lower reading arm resulted in approximately 15% of women and men being misclassified as non-hypertensive at a threshold of BP ≥ 140/90 mmHg due to false negatives.

In future studies, the predictive value of sex-stratified IAD should be assessed for cardiovascular events and death to verify its potential as a screening tool in population-based CVD screening.

### Electronic supplementary material

Below is the link to the electronic supplementary material.

Supplementary Material 1

Supplementary Material 2

## Data Availability

In accordance with Danish law and for data protection purposes, the datasets analysed for this study are not publicly available. However, the datasets are available from the corresponding author on reasonable request, pending data transfer approval by the Danish Data Protection Agency.

## Abbreviations

AAA: Abdominal aortic aneurysm
ABI: Ankle-brachial index
ACE: Angiotensin-converting enzyme
BP: Blood pressure
BMI: Body Mass Index
CP: Carotid plaque
COMPASS: Cardiovascular Outcomes for People Using Anticoagulation Strategies
CPR: Civil registration number
CT: Computed tomography
CVD: Cardiovascular disease
HbA1c: Glycated haemoglobin
IAD: Interarm blood pressure difference
LEAD: Lower extremity arterial disease
MACE: Major adverse cardiovascular events
REDCap: Research Electronic Data Capture
VISP: Viborg Screening Program

## References

[1] Visseren FLJ, Mach F, Smulders YM, Carballo D, Koskinas KC, Bäck M (2021). 2021 ESC guidelines on cardiovascular disease prevention in clinical practice. Eur Heart J.

[2] Magnussen C, Ojeda FM, Leong DP, Alegre-Diaz J, Amouyel P, Global Cardiovascular Risk Consortium (2023). Global effect of modifiable risk factors on Cardiovascular Disease and Mortality. N Engl J Med.

[3] Unger T, Borghi C, Charchar F, Khan NA, Poulter NR, Prabhakaran D (2020). 2020 International Society of Hypertension Global Hypertension Practice Guidelines. Hypertension.

[4] Mancia Chairperson G, Kreutz Co-Chair R, Brunstrom M, Burnier M, Grassi G, Januszewicz A (2023). 2023 ESH guidelines for the management of arterial hypertension. The Task Force for the management of arterial hypertension of the European Society of Hypertension Endorsed by the European Renal Association (ERA) and the International Society of Hypertension (ISH). J Hypertens.

[5] Williams B, Mancia G, Spiering W, Agabiti Rosei E, Azizi M, Burnier M (2018). 2018 ESC/ESH guidelines for the management of arterial hypertension. Eur Heart J.

[6] Clark CE, Warren FC, Boddy K, McDonagh STJ, Moore SF, Goddard J et al. Associations Between Systolic Interarm Differences in Blood Pressure and Cardiovascular Disease Outcomes and Mortality: Individual Participant Data Meta-Analysis, Development and Validation of a Prognostic Algorithm: The INTERPRESS-IPD Collaboration. Hypertension (Dallas, Tex: 1979). 2021;77:650 – 61. 10.1161/HYPERTENSIONAHA.120.15997. 10.1161/HYPERTENSIONAHA.120.15997 PMC7803446 33342236

[7] Clark CE, Warren FC, Boddy K, McDonagh STJ, Moore SF, Teresa Alzamora M (2022).

[8] Heneghan C, Perera R, Mant D, Glasziou P (2007). Hypertension guideline recommendations in general practice: awareness, agreement, adoption, and adherence. Br J Gen Practice: J Royal Coll Gen Practitioners.

[9] Høgh A, Lindholt JS, Søgaard R, Refsgaard J, Svenstrup D, Moeslund N-J (2023). Protocol for a cohort study to evaluate the effectiveness and cost-effectiveness of general population screening for cardiovascular disease: the Viborg Screening Programme (VISP). BMJ Open.

[10] Dahl M, Lindholt J, Søgaard R, Refsgaard J, Svenstrup D, Moeslund N (2023). Relevance of the Viborg Population-based Screening Programme (VISP) for cardiovascular conditions among 67-year-olds: attendance rate, prevalence and proportion of initiated cardiovascular medicine stratified by sex. Eur J Vasc Endovasc Surg.

[11] Danish Health Authority. The Danish National Health Survey 2023 [ https://www.danskernessundhed.dk/.

[12] Schmidt M, Pedersen L, Sorensen HT (2014). The Danish Civil Registration System as a tool in epidemiology. Eur J Epidemiol.

[13] World Health Organization (2020). WHO technical specifications for automated non-invasive blood pressure measuring devices with cuff.

[14] Brady TM, Charleston J, Ishigami J, Miller ER 3rd, Matsushita K, Appel LJ. Effects of different Rest Period durations prior to blood pressure measurement: the best Rest Trial. Hypertension. 2021;78:1511–9. 10.1161/HYPERTENSIONAHA.121.17496. 10.1161/HYPERTENSIONAHA.121.17496 34601959

[15] Singh S, Sethi A, Singh M, Khosla S (2015). Prevalence of simultaneously measured interarm systolic blood pressure difference and its clinical and demographic predictors: a systemic review and meta-analysis. Blood Press Monit.

[16] Schwartz CL, Clark C, Koshiaris C, Gill PS, Greenfield SM, Haque SM (2017). Interarm difference in systolic blood pressure in different ethnic groups and relationship to the White Coat Effect: a cross-sectional study. Am J Hypertens.

[17] Clark CE, Taylor RS, Shore AC, Campbell JL (2016). Prevalence of systolic inter-arm differences in blood pressure for different primary care populations: systematic review and meta-analysis. Br J Gen Pract.

[18] Clark CE, Campbell JL, Powell RJ, Thompson JF (2007). The inter-arm blood pressure difference and peripheral vascular disease: cross-sectional study. Fam Pract.

[19] Methre S, Jayakumar R, Methre T, Joshi P. Correlation of interarm blood pressure difference with family history of hypertension, anthropometric parameters, and mean arterial blood pressure in normotensive people. Nat J Physiol Pharma Pharmacol. 2020;11. 10.5455/njppp.2021.10.08210202026082020.

[20] Clark CE, Taylor RS, Butcher I, Stewart MC, Price J, Fowkes FG (2016). Inter-arm blood pressure difference and mortality: a cohort study in an asymptomatic primary care population at elevated cardiovascular risk. Br J Gen Pract.

[21] Das S, Iktidar MA, Das J, Chowdhury F, Roy S. Inter-arm blood pressure difference as a tool for predicting coronary artery disease severity. Open Heart. 2022;9. 10.1136/openhrt-2022-002063. 10.1136/openhrt-2022-002063 PMC9379529 35961695

[22] Mehlsen J, Wiinberg N (2014). Interarm difference in blood pressure: reproducibility and association with peripheral vascular disease. Int J Vasc Med.

[23] Verberk WJ, Kessels AGH, Thien T (2011). Blood pressure measurement method and inter-arm differences: a Meta-analysis. Am J Hypertens.

[24] Arnett DK, Tang W, Province MA, Oberman A, Ellison RC, Morgan D (2005). Interarm differences in seated systolic and diastolic blood pressure: the Hypertension Genetic Epidemiology Network study. J Hypertens.

[25] Gopalakrishnan S, Savitha AK, Rama R (2018). Evaluation of inter-arm difference in blood pressure as predictor of vascular diseases among urban adults in Kancheepuram District of Tamil Nadu. J Family Med Prim Care.

[26] Gbaguidi GN, Kaboure A, Houehanou YC, Amidou SA, Houinato DS, Aboyans V, Lacroix P (2022). Inter-arm difference in systolic blood pressure: prevalence and associated factors in an African population. PLoS ONE.

[27] Johansson JK, Puukka PJ, Jula AM (2014). Interarm blood pressure difference and target organ damage in the general population. J Hypertens.

[28] Kranenburg G, Spiering W, de Jong PA, Kappelle LJ, de Borst GJ, Cramer MJ (2017). Inter-arm systolic blood pressure differences, relations with future vascular events and mortality in patients with and without manifest vascular disease. Int J Cardiol.

[29] Um SW, Ohle R, Perry JJ (2018). Bilateral blood pressure differential as a clinical marker for acute aortic dissection in the emergency department. Emerg Med J.

[30] Clark CE, Taylor RS, Shore AC, Ukoumunne OC, Campbell JL (2012). Association of a difference in systolic blood pressure between arms with vascular disease and mortality: a systematic review and meta-analysis. Lancet.

[31] Ma W, Zhang B, Yang Y, Qi L, Meng L, Zhang Y, Huo Y (2017). Correlating the relationship between interarm systolic blood pressure and cardiovascular disease risk factors. J Clin Hypertens (Greenwich).

[32] Qadura M, Syed MH, Anand S, Bosch J, Connolly S, Aboyans V (2023). The predictive value of interarm systolic blood pressure differences in patients with vascular disease: sub-analysis of the COMPASS trial. Atherosclerosis.

[33] Karmali KN, Persell SD, Perel P, Lloyd-Jones DM, Berendsen MA, Huffman MD (2017). Risk scoring for the primary prevention of cardiovascular disease. Cochrane Database Syst Rev.

[34] Matthews S, Cook S, Clayton T, Murray S, Wynne R, Sanders J (2023). Factors affecting women’s participation in Cardiovascular Research: a scoping review. Eur J Cardiovasc Nurs.

[35] Duprez DA, Jacobs DR, Andrews LIB, Brumback LC, Denenberg JO, McClelland RL (2023). Inter-arm systolic blood pressure difference: non-persistence and association with incident cardiovascular disease in the multi-ethnic study of atherosclerosis. J Hum Hypertens.

[36] Clark CE, Horvath IA, Taylor RS, Campbell JL (2014). Doctors record higher blood pressures than nurses: systematic review and meta-analysis. Br J Gen Pract.

