# Supplementary material for: Prevalence of interarm blood pressure difference is notably higher in women; the Viborg population-based screening program (VISP)
Source: BMC Public Health. 2024 Jul 12;24:1868. doi: 10.1186/s12889-024-19388-8 (PMC11245839; doi:10.1186/s12889-024-19388-8)
Supplement: Supplementary file 1 — Supplementary Material 1 [file 12889_2024_19388_MOESM1_ESM.pdf]

VIBORG KOMMUNE

sundhedscenter VIBORG

Health Examination Survey

for cardiovascular diseases and diabetes
67-year-olds living in Viborg Municipality

Name ______________________________

Civil registration number (CPR) ______________________________

Telephone number ______________________________

What is your height (cm) ______________________________

What is your weight (kg) ______________________________

MEDICATION:

Do you take any medication? Yes ☐ No ☐

If yes, please state the name of the medication or bring your medication list

DISEASES:

For each of the following disease, please indicate whether you currently have it or have had it in the past.

	No, I have never had	Yes, I have	Yes, I have previously had
Diabetes	☐	☐	☐
High blood pressure	☐	☐	☐
Heart attack	☐	☐	☐
Angina pectoris	☐	☐	☐
Stroke	☐	☐	☐
Migraine or frequent headaches	☐	☐	☐
Atrial fibrillation	☐	☐	☐
Peripheral artery disease	☐	☐	☐
Abdominal aortic aneurysm	☐	☐	☐
Chronic obstructive pulmonary disease	☐	☐	☐
Canser	☐	☐	☐

Do you have a chronic disease, long-term effects from injury, disability, or any other prolonged condition?

By "chronic," we mean lasting at least 6 months Yes ☐ No ☐

If yes, please specify which one ____________________

Do you have a mental illness? Yes ☐ No ☐

If yes, please specify which one ____________________

LIFESTYLE

How would you rate your overall dietary habits?

Very healthy	Healthy	Fairly healthy	Unhealthy	Very unhealthy
☐	☐	☐	☐	☐

Do you smoke?

Yes	No, I stoped	No, I have never smoked
☐	☐	☐

How many units of alcohol do you typically consume in a week? ____________

1 drink: 1 beer, 1 glass of wine, 1 drink/cocktail, 1 snaps/shot and 1 alcoholic cider
6 drinks: 1 bottle of wine
20 drinks: 1 bottle of spirits

How would you characterize your leisure-time physical activity when reflecting on the past year?

☐ Reading, watching television, or another sedentary activity.

☐ Walking, cycling, or other light exercise for at least 4 hours per week.

☐ Engaging in sports or performing heavy gardening or similar activities for at least 4 hours per week.

☐ Exercising intensely and participating in competitive sports regularly several times a week.

Other

Are you employed at the moment? Yes ☐ No ☐

If yes, how many hours per week do you work? ____________

Do you ever find yourself alone when you would rather be with others?

Yes, often ☐ Yes, occasionally ☐ Yes, but rarely ☐ No ☐

THANKS

for your reply
