# Supplementary material for: Prevalence of interarm blood pressure difference is notably higher in women; the Viborg population-based screening program (VISP)
Source: BMC Public Health. 2024 Jul 12;24:1868. doi: 10.1186/s12889-024-19388-8 (PMC11245839; doi:10.1186/s12889-024-19388-8)
Supplement: Supplementary file 2 — Supplementary Material 2 [file 12889_2024_19388_MOESM2_ESM.docx]

**Supplementary Material** **2.**

**Figure S1. IAD prevalence, in total and in 10 mmHg intervals, stratified by sex.**


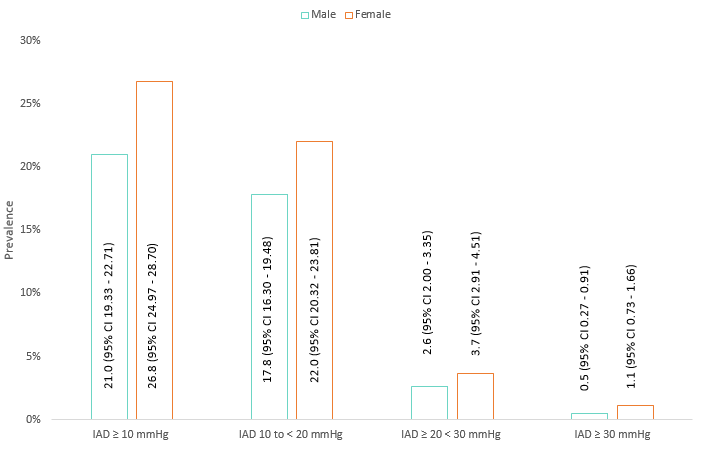


Abbriviation: IAD, interarm blood pressure difference.

**Figure S2. Venn diagram displaying coexistence of IAD ≥ 10 mmHg and BP at 140/90 mmHg or 160/100 mmHg thresholds, stratified by sex.**


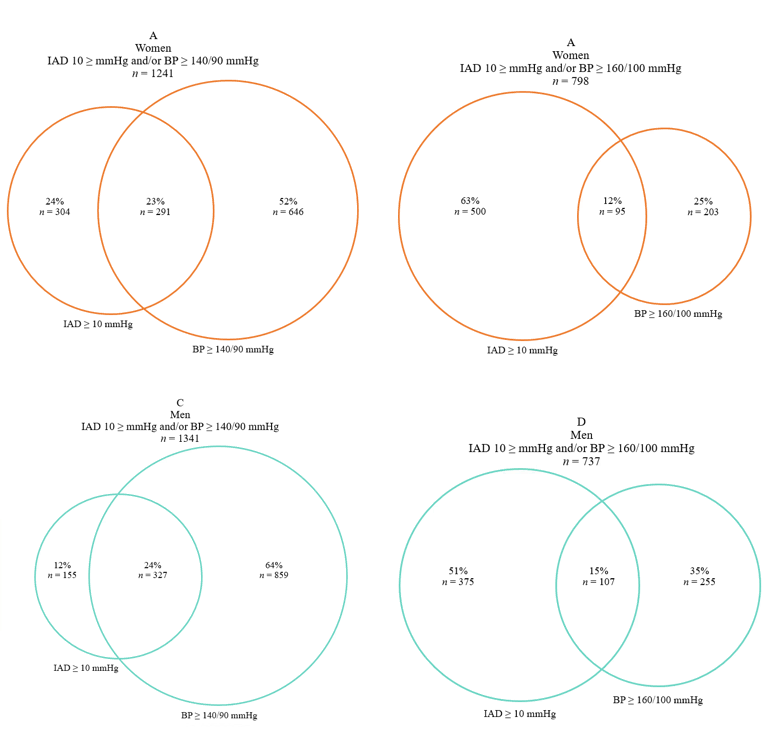


Abbreviations: BP, blood pressure; IAD, Interarm blood pressure difference.

Percentages are for those with IAD and/or raised BP (women, A and B; men C and D).
